# Avoidant Coping of the Decision-Making Process on the Location of Care in Old Age: A Possible Conspiracy of Silence?

**DOI:** 10.3390/ijerph182412940

**Published:** 2021-12-08

**Authors:** Gema Serrano-Gemes, Isabel Gil, Adriana Coelho, Rafael Serrano-del-Rosal

**Affiliations:** 1 Institute for Advanced Social Studies-Spanish National Research Council (IESA-CSIC), 14004 Córdoba, Spain; rserrano@iesa.csic.es; 2 Maimonides Biomedical Research Institute of Cordoba (IMIBIC), University of Cordoba (UCO), Reina Sofia University Hospital (HURS), 14004 Córdoba, Spain; 3 Escola Superior de Enfermagem de Coimbra, Unidade de Investigação em Ciências da Saúde: Enfermagem (UICISA: E), 3004-011 Coimbra, Portugal; igil@esenfc.pt (I.G.); adriananevescoelho@esenfc.pt (A.C.)

**Keywords:** conspiracy of silence, pact of silence, decision-making, older people, location of care, qualitative

## Abstract

The conspiracy of silence is extremely important due to both its high incidence and its consequences. This process usually occurs in situations of palliative care, or death; however, this concept is also mentioned in the literature linked to other contexts. Therefore, our objective was to study whether the conspiracy of silence may be extrapolated to the context of decision-making on the location of care in old age. To this end, we first analyzed the in-depth semi structured qualitative interviews conducted with older people, caregivers, and professionals, about decision-making on the location of care in old age. Subsequently, a comparative analysis was performed between the basic elements of the conspiracy of silence and this decision-making. Our findings revealed an avoidance process developed by all three groups. Furthermore, this decision-making presents similarities with the conspiracy of silence in the process of avoidance coping and denial that is developed. However, there are significant differences, as information is not withheld from the older person, who has an active attitude in the process of avoidance. Decision-making on the location of care in old age does not exactly match the conspiracy of silence process, but it does seem to correspond to a pact of silence.

## 1. Introduction

The conspiracy of silence is described in scientific literature as an agreement among family, friends, and/or health professionals to modify the information given to a patient; this agreement may be explicit or implicit [1,2,3,4]. The objective of this behavior is to hide the diagnosis, prognosis, and/or the seriousness of the situation [1,2,3], and, as pointed out by Vergara Lacalle [5], the most crucial information seems to be the one dealing with prognosis.

This phenomenon also presents at different levels, as it may be partial or total [6,7]. The partial level occurs when the patient knows their diagnosis, but not their prognosis [6,7]. While the total level the patient does not know either [6,7], either if they deduce their actual state or not [6]. In addition, this phenomenon may be adaptive or maladaptive [1,6,8,9]. If it is adaptive, it is the patient who, voluntarily, avoids, does not talk about, and/or denies the information [1,6,8,9], implying that they do not want to know the diagnosis or prognosis, regardless of the family member’s attitude [8]. If it is maladaptive, even if the patient wants to have information about their state, the family [1,6,9] or health professionals [6,9] prevent them from accessing such information. 

Thus, according to Bermejo et al. [8], this second type may occur in two different ways. It may be motivated by the family member’s attitude, because they want to protect the patient or to protect themselves (as long as the patient is asking or not showing an attitude against the information process) [8]. Or it not be motivated by the family member’s attitude and, in principle, supported by health professionals, where the family member and the patient have an attitude that does not seem to be against receiving information [8].

The conspiracy of silence phenomenon is a process of great interest. In fact, the recent integrative literature review carried out by Costa Machado et al. [10] about the conspiracy of silence in palliative care informed about the importance of this phenomenon, among other reasons, due to its high occurrence in patients receiving palliative care [10]. It is also noteworthy because of the consequences it brings about, since, unfortunately, it is not innocuous. This phenomenon causes diverse serious problems affecting not only those involved patient [1,2,3,6,9,11,12,13,14,15], but also the family that is generating the conspiracy of silence [1,2,3,4,6,9,11,12,13,14], the professionals assisting them in it [1,2,3,9,11,12,16], and even the health system itself [11,12].

Therefore, this phenomenon provokes an awkward and painful situation for patient, family, and professionals [9], and, more precisely, it has a tragic impact on family relationships, since this process leads to systematic deceiving and hypocrisy, undermining previous solidarities [13].

In view of the literature consulted before writing this paper, the context where the conspiracy of silence occurs would be situations where a patient is receiving palliative care, or matters linked to death; Ibañez-Masero et al. [4] points out that this is a process that takes place at the end of life. Nevertheless, this concept is also mentioned in the literature linked to other contexts, such as: the conspiracy of silence applied to alcohol abuse in doctors [17], in the participation of doctors in legal proceedings to assess the quality of other doctors’ work [18], when it comes to not talking about eating disorders due to cultural backgrounds [19], linked to not talking about human immunodeficiency virus/acquired immunodeficiency syndrome [20], linked to not talking about hypertension treatment [21].

Due to all this, this paper will focus on analyzing, through an in-depth study, whether this conspiracy of silence process may be extrapolated to other context, specifically, to a previous situation given before care at the end of life—older people care when they start needing help. This context has been chosen because thanks to the qualitative interviews conducted with three different population groups, it has been seen how there is a process of avoidance of decision-making on the location of care in old age. Its possible similarity and connection to the conspiracy of silence process is also mentioned.

In view of these findings, a systematic review of the literature was conducted to see if there were any studies that related the conspiracy of silence to the decision about the location of care in old age [22]. However, this review did not retrieve any publication dealing with the abovementioned topics of interest [22]. Despite this, a previous wider review [23,24,25,26], provided data revealing that, a priori, in certain studies of the literature, families seem to hide the decision-making process from the older people [24]. In that review, it was observed that, in certain cases, family members make the decision without the older people, informing them whenever the final decision is already made [24]. 

Due to all of this, this paper’s objective is to study whether the conspiracy of silence process may be extrapolated to the context of decision-making on the location of care in old age.

## 2. Materials and Methods

### 2.1. Design

Information obtained from a qualitative study conducted through semi-structured in-depth interviews. 

### 2.2. Period of the Study

The in-depth interviews were conducted in two phases. The object of study in the first one was health and social care professionals, and it was carried out from 21 February 2018, to 6 November 2018. The second phase focused on the older people and their caregivers, from 22 May 2019, to 26 July 2019.

### 2.3. Field of Study

The qualitative study was entirely carried out in the healthcare district of the south of Spain, more precisely, in two different healthcare areas: an urban area (three health centers and a nursing home), and a rural area (a health center, a doctor’s office, and a nursing home located near the doctor’s office).

In addition, different population groups of interest were in this study, both in the professional and in the family spheres. In the former sphere, doctors, nurses, social workers, and management professionals were interviewed. In the latter group, caregivers and older people were interviewed, both living at home or in a nursing home.

### 2.4. Inclusion and Exclusion Criteria

To be included, professionals had to be working in the areas to be studied, with at least 2–3 years of professional experience, and have a managerial position or be doctors, nurses, or social workers. The latter three, throughout their experience, had to have been in contact with a patient facing the decision of staying at their home or moving to a nursing home.

We sought professionals with experience not only in clinical work, but also in management functions. We wanted to know whether the latter had a different opinion from that of exclusively clinical professionals. In addition, due to the functioning of the Spanish public health care system (the context in which the study was carried out), we wanted to have the opinions of professionals who were closer to the organization and management of resources.

Caregivers had to be somehow in charge of an older person (a person who is 65 or older, living at home or in a nursing home, who had faced the decision of staying at home or moving to a nursing home), and they had to remember the experience of making the decision. Although sex was not an inclusion/exclusion criterion, of the ten caregivers interviewed, nine were women. This is in line with the Spanish caregiving situation.

The older people had to be 65 or older, had to have faced the decision of staying at their home or moving to a nursing home, had to live at the home/a nursing home, and had to remember the experience of making the decision.

People with cognitive impairment, hearing impairment, memory problems and/or disorientation were excluded. In addition, also excluded were those who simply did not wish to participate.

These exclusion criteria were studied in two ways: the knowledge and experience of the professionals who provided us with the interviews (the medical diagnoses of the older people as well as the assessment of their capabilities) and/or the criterion of the principal investigator based on the possibility of developing the interview in an appropriate way according to the capabilities and condition of the person, which were not only limited to their cognitive state, but also emotional and physical.

### 2.5. Sample Size and Sampling Technique

A purposive sampling was performed, thus finding different specific profiles and key informers. Therefore, the participants were carefully and intentionally selected, based on their possibility to provide information of interest. Descriptive information about the study participants can be found in Table 1.

Therefore, and according to the purposive sampling, there was no preestablished number of participants [27], but information continued to be gathered until the discourse was saturated.

The sampling was performed in two phases: first, accessing the professional context, and, later, the family context.

### 2.6. Data Collection Procedure

In-depth semi-structured interviews were conducted.

All the interviews were conducted by the main researcher. In order to ensure the informed consent, before starting the interviews, we confirmed that the individuals complied with the inclusion criteria, and that they were informed and all the doubts about the study had been answered in an appropriate manner, then accepting to take part and signing the informed consent form.

All the interviews were recorded in audio and transcribed, ensuring the reliability and consistency of the data gathering. A total of 49 interviews in total were conducted (25.73 h of recording time, with an average duration of 31.5 min, and 486 pages of transcript). 

The transcripts were originally performed and analyzed in Spanish, the language used by the interviewees. However, for the purposes of this article, they have been translated into English by a professional translator and afterwards, back-translated to check the accuracy of this process. 

The information obtained has been triangulated at several levels: among the results of the different populations studied, and with the available bibliography and/or conceptual framework.

### 2.7. Data Analysis

First, the data analysis was carried out manually using the constant comparative method [28] from Glaser and Strauss’ grounded theory [29].

Second, once the categories of results were obtained and analyzed, a comparative analysis was made between the decision-making process regarding the location of care and the conspiracy of silence. To this end, the basic elements of comparison were identified. They have been selected by the authors according to the literature review that was previously conducted in order to carry out this study. They are the following: participants involved in the process, affected population, the problem that needs to be solved, the coping processes, the reasons to undertake it, and the conflicts created throughout the process.

The result of the first analysis is shown in the findings section. While the results of the second analysis are shown in the discussion section due to their comparative nature.

### 2.8. Declaration of Ethics

The research project of the complete doctoral thesis where this study is framed was approved by the Research Ethics Committee of Córdoba (File No. 265, Ref. 3533). 

This research complies with current regulations on personal data protection and the regulatory framework of reference for research projects in Spain and the European Union, maintaining at all times the standards for good clinical practice and the fundamental ethical principles established for research by the 1964 Declaration of Helsinki, by the World Medical Association, as well as its subsequent amendments, and by the 1996 Council of Europe’s Convention for the Human Rights and the Biomedicine.

## 3. Findings

**Figure 1:**
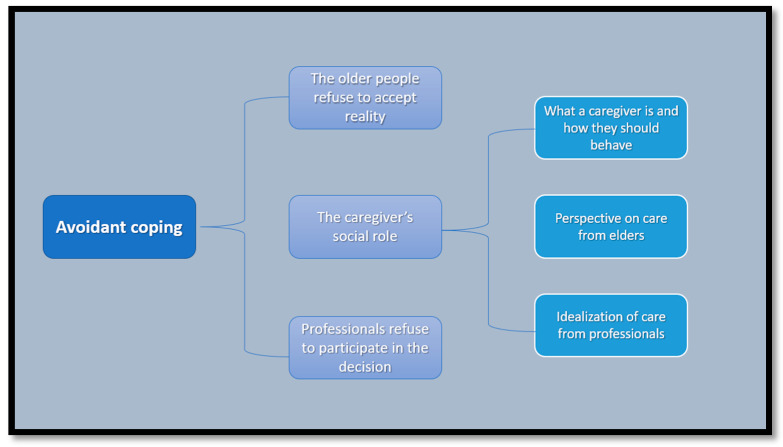
Summary of findings categories. Source: prepared by the authors.

This article shows the most important and interesting matters regarding to the avoidance process developed by the different participants. Thus, different categories of results related to the avoidance process emerge. These main categories can be seen in summary form in Figure 1 and in detail and developed form in the text below.

The results in this category have revealed how the decision on location of care in old age takes place under an avoidance coping process, in which somehow all the studied population groups take part and are involved.

In general, the study shows how thinking of, talking about, and facing the issue of care is avoided, denying the need to make a decision, avoiding becoming involved and, in short, making changes in one’s way of life in a way that is not calm and planned, but reacting to the events as they occur.

This way of avoiding the need of making a decision was compared by one of the interviewed directors of a health center to the conspiracy of silence. This professional pointed out how everyone who surrounds the older person, and even the older people themselves, know that there is a problem, but they do not want to talk about it, even if eventually this will become unapproachable due to the circumstances.

This is because, seemingly, no one wants to be the one to take the first step to make a decision that, for better or worse, will make changes in the option that is wanted and preferred by everyone, which is staying at home (with, in any case, the help of certain home care). Usually, the decision that is most often postponed is moving to a nursing home.

*“The decision makes itself, this is just like… the conspiracy of silence, I don’t know if you are familiar with the conspiracy of silence in palliative care, I’ve got cancer and I know it but I don’t say a word, my family knows it and they don’t tell me anything, and if I don’t know about it my family doesn’t tell me either, but there is a feeling there that things are not ok but no one wants to talk about it. We stretch care at home so much, but we know that the time will come, but we don’t talk frankly about that moment […] You see family tensions, grandpa is the problem, the children sit down to talk about the problem of grandpa, those issues are not dealt with calmly and quietly, […] so the decision is taken in a wrong way, at a wrong time, causing frictions, and grandpa is in the middle, probably many of them won’t even ask him if he wants to leave, or not, or what should we do, this is like taboo today […] and I can tell you that 90% of the times it is done like that, in a somehow traumatic way, the transition is not normal and natural, […]” (Managerial position: Nurse. Man)*

This is why even when the circumstances make the need to make a decision clear, the situation is maintained in time, obviously because caregivers keep on providing care. This decision is avoided, then, until the caregiver can no longer cope with caregiving in any way, thus making the need to make a decision about how to meet the older person’s needs for care unavoidable.

*“Well, look, I remember one night, [...] I was raising him up, I had a drop in blood pressure, I lost consciousness a little bit, [...] I had him, I think it was about twenty minutes or so, leaning on a, on the bedside table, because I was on the floor, unconscious, I lost consciousness, and of course, I said, well, what if I get sick? I saw it as the worst, and after that I started to think, I said no, it can’t be, I was exhausted, I wasn’t sleeping at night... it was horrifying”. (Caregiver. Woman)*

Regarding the avoiding and reactive nature of decision-making, it is important to point out that, due to how the system and the public care services are structured in our area, it is almost impossible to make decisions in a planned manner. This occurs because it is difficult, if not impossible, to have access to these public resources when a person makes a decision. Quite the opposite, these resources are only available when it is possible for the system, and there are two main problems. First, the time that it takes for public authorities to give an answer. Therefore, on many occasions, when an answer is given, the needs of the applicant have changed, usually increasing. Secondly, the scarce resources that are available are only accessible to some extent for people with high needs for care.

*“The system itself, let’s say, does not encourage things to be done well, so in many cases the person is forced by time, [...] he/she has to decide in a hurry, because... it has to be sent now, [...] the system itself ends up forcing decisions to be made quickly, [...]. It’s that, because if once the person makes the decision, then there is a change of decision, there is a regret, no, in the end I don’t want this, it is administratively, so to speak, bureaucratically damaging, the times are duplicated, so to speak, so, the person also has this fear, which is that it seems as if the decision, there is not this capacity of well I am going to try and then come back, it is not so easy in reality, because if later a change has to be made, this leads to a lot more paperwork that has to be taken up again. [...]” (Social Worker. Man)*

Finally, apart from what has already been expounded, it is important to mention the avoidance strategies in decision-making developed by each of the studied population groups, which are explained in short in the following section.

### 3.1. Avoidance in Older People: Refusing to Accept Reality

Our results have shown how losing the capacity to take care of themselves makes older people de facto lose the power to decide. This implicitly means for them that the fact of accepting that it is necessary to make a decision is accepting not only their own loss of capacity, but also of much of their autonomy.

This is why, throughout our results, in some cases, the older people’ self-perception is quite distorted, having an untrue view of reality, not considering the changes undergone or their own capacities. 

The older people, through this self-delusion process, modify their discourse as they lose capacities, modifying concepts and definitions to this end, so that they adapt to their own health condition and independence at all times. In this way, many older people who live at home point out that they can stay there because they are able to live independently (or almost independently). However, what it means to live independently varies according to the person: for some people, it means being able to get up and get into bed and to wash themselves up, while for others it means going outside, or preparing their meals, etc.

*“Well, I don’t do anything, I don’t do, they even bring me the food, because I can’t anymore, can’t do things in the kitchen and that kind of thing, just my coffee in the morning and that’s it. […] I can no longer do as I did four or five or six or ten years ago, that’s nonsense, the years don’t pass in vain, you notice things, but, she sees that I’m well, I can walk and I can do everything on my own, I can clean myself, I can go to bed and get up by myself, I do everything on my own.” (Older person. Woman)*

This distorted self-perception is maintained because these older people are able to lead a rather independent life because they have more or less help, or because they have refused to include certain activities or functions in their lives. Thus, on some occasions, the older people seem to omit or not value the activities that they are not able to do, and that other people perform for them (either through public or private aids, or their own families), so they understand that they are still independent, without accepting that they are actually in a state of semi-independence which may eventually become complete dependence.

*“And what does your daughter help you with? I mean, what do you need help with?] I don’t need any help, come on, because right now I have a nerve problem and dizziness, and my, and then my legs are very, very delicate too, my wife’s knees hurt, [...] we are both, we are delicate, but we are fine […]” (Older person. Man)*

The distortion in self-perception appears mainly in people who live with their caregivers. These people, apart from not accepting their own health condition, do not accept either that the caregivers who take care of them are also burnout, making it impossible for them to continue devoted to care indefinitely. However, this distorted view is not always so extreme, but there are older people who realize that their health conditions have worsened, even if they mostly believe that their situation will remain in time and that they will be able to go on living the way they are (or, in any case, with minor changes). Thus, this denial of reality seems to be closely linked to the fact that decision-making is avoided and delayed, since, in a more or less active way, the older people refuse to recognize their deterioration and/or their caregivers’, not showing that a decision will have to be made sooner or later, trying to delay making decisions that affect their lifestyles as much as possible.

*“I think that she is sacrificed for me, but I am not going to be taken care of by someone like her... [...] I have already told you before that I think she is not well, but, as long as she can, of course I prefer that she take care of me to another person. [...] but I know that one day has to get there, I have taken her to three paid doctors, bone surgeons, and all three have agreed that she has a ninety percent chance of remaining in a wheelchair [...] how am I going to operate on her? how is she going to operate? [...] From what the doctors say yes, but I know she is very active, and no, hum hum, she is going to be holding on until the last minute, so she, she is going to hold on because she, I told you, she is in the, in the pain unit and they are looking at her to see what they can do, [...]” (Older person. Man)*

### 3.2. Avoidance in Caregivers: The Caregiver’s Social Role

Caregivers also refuse to accept the changes undergone by the persons they are taking care of, denying that they require more and more time and abilities from them. Caregivers also de facto deny their limited capacity to continue in their role as caregivers indefinitely. This denial does seem to be conscious in most cases, as opposed to that of older people.

*“I say that as long as I can be here, I will be here... But, tomorrow I don’t know... [....] Tomorrow I will see what I have to do as I go along, because I, because if he worsens like his mother did I can’t, because when I took care of his mother I was twenty years younger, but now I cannot, because of my back and, and that, the years do not pass in vain, I have had surgery on my womb, I have had surgery on my eyesight, I have had surgery on my hip and, and I cannot, I cannot, [...] because as long as I can, I am going to take care of him and I am going to keep an eye on him, but of course, when I fell yesterday, for example, I said, my God of my soul, may nothing happen to me, I said, because if not, what am I going to do? [...]” (Caregiver. Woman)*

Refusing to provide care, or giving it up, seems to be a serious conflict not only with their social and family context, but also with their own values, which makes them deny the situation until reality sets in, that is, when a point of no return is reached in the health condition of older people and/or the capacity of caregivers to meet the needs of older people. Taking this attitude, they avoid having to make a decision that would break away with the ideal of care.

Caregivers, despite being aware of their exhaustion, insist on continuing taking care of older people until they cannot take it any longer. This seems to point out that they do not want to be the ones to give up, but, instead, they prefer to have an external reason make them give up on care, thus freeing them from the responsibility. Therefore, it seems that there has to be a sound reason to give up on care, a reason that obliges them to do so without any plausible alternative, since this would socially justify caregivers not only before society in general, but before their own family, the person they are taking care of, and themselves, proving that they have fulfilled their role. This is why institutional care, rather than an option to be chosen, is the option to turn to when all the other options have been exhausted.

In short, caregivers seem to be affected by the social construct of what care should be like; this influence may be broken up into three main elements.

#### 3.2.1. Their Own Conceptualization of What a Caregiver Is, and How They Should Behave

When a family is in charge of taking care of an older person, this does not seem to be an election as such, chosen in a reasoned, free way, but it seems to be a socially accepted imposition. This mandate of care does not seem to be lived traumatically, but generally accepted, as it is seen as something cultural and traditional. However, not every individual in a family seems to have the same obligation to provide care. The weight of this obligation is shaped by two variables: sex and kinship. Thus, the responsibility of care seems to be given on the grounds of sex, while the level of involvement seems to be affected by the degree of kinship of the caregiver. There are two main levels of kinship: close family (spouses and daughters) and extended family (sisters, nieces, and other relatives).

*“No, I don’t have another one, he is my husband, I don’t have another one. [...] No, I haven’t thought about it because, man, I don’t put my husband in a nursing home, unless he gets very bad, very bad, very bad and I need a woman day and night, but how am I going to put my husband in the nursing home, after all he has fought and worked for his children? [...] No, no one has told me anything, he is my husband, I have to take care of him and that’s it” (Caregiver. Woman)*

#### 3.2.2. Perspective on Care from Older People

The older people pointed out differences in how care was managed when they were young and today. In general, the older people explained how families, and women specifically, were the ones tending the home, not having to look for help elsewhere, while today families do not want to provide care. Some older people understand that this is caused by today’s social circumstances. Therefore, they talk about how years ago the role of women was to stay at home doing household chores and also taking care of older people and children. They also point out that today it is necessary for men and women to work outside the home, viewing this situation as a worsened scenario within the family.

*“[…] because society has changed so much, you are very young and you, it’s not, you haven’t seen that, but look, how families worked back in the day, […] normally the last one, or the last daughter to get married would stay at the parents’ home and so this person who lived in the house would inherit it, so they would kill two birds with the same stone, taking care of the parents and having a house, so that’s, people don’t want that anymore, not even if you pay them, if you give them money, nobody wants that, […] and that doesn’t make people better or worse, but things have changed, society has changed and that’s it, don’t even think about it, back in the day only men worked and women were taking care of the children […] but that was what it was, how it worked and that’s it, everyone would accept that, but you can’t have that today, first of all, there’s no one, very few people, who make enough money to have their wife at home doing nothing and buy a three hundred thousand euro condo, today both of them have to work or things don’t work out, […]” (Older person. Man)*

Therefore, the interviewed older people may be classified into two groups: those who accept the new situation and those who lament/reproach it. The first group understands that this social change is nobody’s fault, but life has simply changed, while the second group, despite acknowledging that their daughters are very busy, feel that they should give them more care and pay more attention to them.

#### 3.2.3. Idealization of Care from Professionals

As for health and social care professionals, some of them point out that family care is not that traumatic, as it is understood as something natural (it has always been similar to that). Therefore, in general, when the family takes charge of the older person, it is seen as something positive, sometimes recognizing that this opinion is based on personal preferences for the future or past experiences.

*“[…] I see older people who are in houses, who well, maybe, to our eyes, are not well cared for because maybe the environment is not beautiful, it is not perfect, but they have a dose of affection, which is obvious, that is, maybe they have a torn sofa, but they are cared for and their grandchildren are around them and it is, they have a nice family environment [...]. When it is taken, when I go to homes and I see decisions, yes, I like it, because although I see that the grandfather may not be so well cared for, but he is fine, I like the fact that the family chooses to have their older person [...]”. (Nurse. Woman)*

In addition, the interviewed professionals considered family care both positively and negatively. Therefore, although they usually recognize that the family is in charge of taking care of the older people and that this care is usually adequate, some professionals have a radically opposite opinion, considering that this care is completely insufficient. They consider that families complain too much and try to abuse the system, asking for too many resources/help, pointing out that the family could be more involved in care, sometimes even assuming that the objective of the family is to stop taking care of the older person for selfish reasons: to have fewer concerns.

*“[...] also sometimes the involvement of the family, I can’t, I can’t, I can’t, I can’t, because I’m sick, I don’t know what, well, we are already entering into, into other value judgments, but, lately I don’t know, sometimes the family could get involved a little more, [...]” (Managerial position: Physician. Woman)*

Nevertheless, the fact that the family takes charge of care does not free them from the judgement of these professionals, since, on some occasions, they even judge the reasons that make the families take on care. Thus, the family’s involvement in the older person’s care seems to be judged by the professionals either if it takes place or not. It is possible that this stems from an idealized conception of older people care.

*“[...] Normally, why do they make this type of decision? Well, the normal thing is that the pension that the older person has is kept by the person who is taking care of them... on many occasions it is because of that, on other occasions because they want to participate in the care of their father and they decide that this is the solution, but in the majority of, [laughs] it is because I am keeping the pension, this month my mother is with me, my mother’s pension is mine, next month it is with my brother, it is with my brother.” (Nurse. Woman)*

### 3.3. Avoidance in Health and Social Care Professionals: Refusing to Get Involved in the Decision

The participation of professionals in this decision-making process is characterized by two elements of interest: avoiding directly getting involved in the decision and professional participation based on their own personal preferences and experiences.

Professionals move away from this decision, as they understand that it belongs to the family sphere and, if it requires professional support, this would be almost exclusively social, considering that, in any case, social workers would be the ones who could have a more active role (even if they understand that they should not be the ones to make a decision either). Health professionals, in general, are reluctant to interfere. Therefore, some are afraid of the consequences it might entail, in some cases, pointing out their refusal to bear someone else’s responsibilities.

*“We… Making that type of decision is complicated and no one wants to make it, I’m being that clear because of the experience that I have here, in, in my office, ok? And I’m not speaking as [Name] the nurse, but I’m speaking, we, eh, we are very reluctant because of the consequences it might entail, I mean, why would I want to, quote unquote, say this if this isn’t my thing, doing my part, which is healthcare, is already enough, ok? I inform them, that’s enough, ok? If someone asks me for a report, I write the report, which is no small thing, look, no small thing, a report about a multipathological patient who has this and that cognitive impairment, and this and that, I’ll send it, but becoming more involved, or stating if I am for or against that patient—it’s not usually done explicitly, it’s not usually done, ok? You are aware that something’s going on there, you inform about it, and then you say now you can ask me for anything, based on that. […]” (Managerial position: Nurse. Man)*

On the other hand, the professional performance of healthcare professionals seems to be guided rather by their personal individual opinions and preferences than by strictly professional guidelines, as they are acting according to their opinions, and they do not know how their colleagues act.

In view of the discourse of healthcare professionals, it appears that there is an evident lack of information, which is not the case in social workers. In fact, the latter mention protocolized systems and procedures that they must follow in order to perform their job properly. However, on some occasions, the other professionals seem to not only be unaware of the procedures followed by their colleagues, but also, as seen clearly in their discourses, there is no routine protocol to follow in their case. 

Therefore, healthcare professionals seem to be guiding their professional performance according to their personal experiences and opinions, usually agreeing to a great extent with the older people and their families’ preferences. 

A synthesis of the most relevant information from our results is shown in Table 2. This information has been used in the comparative analysis shown in the following discussion section.

## 4. Discussion

The process of making decisions on the location of care presents an avoidant coping process developed by all the population groups studied. Each of these groups develops a different avoidance strategy. Thus, the older people deny reality. Caregivers deny reality while being influenced and pressured by both internal and external factors. While professionals avoid involvement in the decision.

In view of these results, the characteristics obtained were compared with the key elements that characterize the conspiracy of silence in order to verify the existing similarities and differences.

In the following paragraphs, each of these elements is developed, setting out, first of all, the relevant information from the literature as regards the conspiracy of silence, followed by a comparison with the results of our qualitative study. A summary of these comparisons may be found in Table 2. 

### 4.1. Participants Involved in the Process

The conspiracy of silence mainly consists of a triangular structure of its participants (patient, family, and professionals) [2,3,9,11,12,16], although other sources in the literature point out the importance of the healthcare institutions [11] themselves, as well as the effect and weight of the socio-cultural context [7,14,30]. The decision on the location of care has the same participants, with this aspect a priori being very similar in both processes.

Nevertheless, there is a fundamental difference as far as the role played by the participants is concerned, which is found in the patient/older person, who is the only one that is different in both processes. While in the conspiracy of silence the patient is the participant that is really affected by the deceiving developed throughout the process, being a passive participant in the other participants’ conspiracy, in the decision on the location of care, however, the older person takes an active role in the process of deceiving and avoiding, similar to the other participants. 

### 4.2. Affected Population

The conspiracy of silence usually occurs on individuals who are considered vulnerable [9,11]: terminal patients [9] or patients with a prognosis of imminent death [11], with physical or psychological problems [9], children [9,11], teenagers [11], or older people [9,11]. Therefore, even if it seems that this is well studied in children, clinical experience seems to point out that it frequently appears in adults [2] too. In fact, more specifically, according to the literature, it seems that age is an important factor to conceal information [7,31]; Villamil Cajoto [7] points out that in palliative care there seems to be a disabling trend mainly among the older people population, which, if linked to the fact that the information about a terminal condition is usually concealed, makes management more difficult [7].

As for the decision on the location of care, as seen in the results of the abovementioned study, the decision affects mainly older people, as the debate focuses on their care. However, it is important to recall how caregivers may also be considered as affected individuals in this process, as avoiding making decisions affects them directly, since they are the ones who have to bear the task of care and to continue doing it. 

### 4.3. The Problem That Needs to Be Solved

The literature about the conspiracy of silence usually deals with situations of palliative care or matters are related with death; Ibañez-Masero et al. [4] point out that this process is something that occurs at the end of life.

However, the data from our study are framed within the context of needing help and care in old age, and, more precisely, making decisions about the location where they will be given.

### 4.4. Coping Processes

In the conspiracy of silence, the involved person’s family is in charge of developing the process, with the help of professionals, taking on a coping strategy based on avoidance and denial of reality [1,2,3,6,14], thus postponing having to face the painful truth [1,2,3,14]. As stated above, the modus operandi is concealing from the patient their real condition, being deceived both by the family and by professionals. 

**Figure 2:**
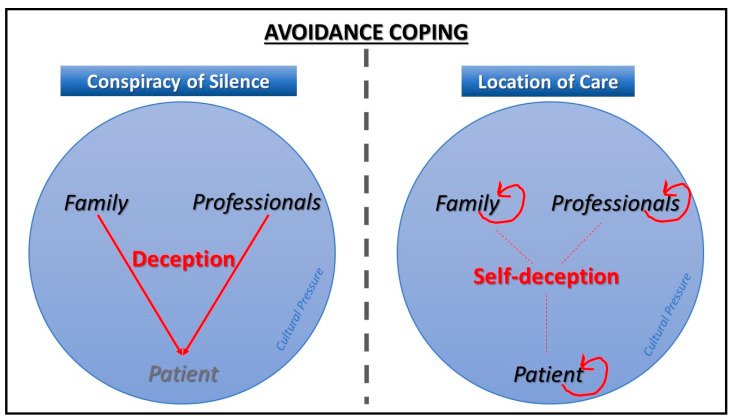
Different types of avoidance coping. Source: prepared by the authors.

Nevertheless, in the case of the location of care, despite sharing the strategy of avoiding and denying reality, there is no concealment of information as such. In addition, as opposed to the conspiracy of silence, it is observed that in this process the family, the professionals, and the older people themselves do the deceiving, not to the others, but to themselves, deceiving themselves. This may be seen graphically in Figure 2.

### 4.5. Reasons to Undetake It

In the literature about the conspiracy of silence, the motives of the different people involved are explained. The involvement of certain people or others depends on, as mentioned above, the type of conspiracy that is carried out, for instance, whether it is adaptative or maladaptative. That is why, in the following paragraphs, and taking into account that the classic conspiracy of silence corresponds to the maladaptative kind [5,14], only the motives of the main active actors in the latter will be included: family and professionals.

The family is usually mentioned in relation to two main types of motives: aspects relating to protecting the patient [1,2,3,4,6,9,11,12,13,14,32], and with self-protection [1,2,3,6,9,11,13,14], whereas professional motives may be classified into three main groups: having difficulties to communicate information [1,2,3,11,14], apparent fear of becoming overinvolved [2,11,16], and having preconceived ideas and images about the way illness is faced by patients [11,33].

Meanwhile, in the case of the decision on the location of care, the main difference is that, as stated above, the older people are active participants in the avoidance process, having their own motives to not want to start the decision-making process. These motives are usually within their denial of not only their loss of capacities, but also their refusal to accept the consequences of care on the caregivers, thus avoiding having to change their lifestyle.

As for the populations of caregivers and professionals, certain similarities have been found in both processes. The first one may be how the social pressure factor influences the caregivers’ motives, making them want to continue giving care, denying their own need to give up this activity, as well as the older person’s condition. This circumstance has a certain connection with the conspiracy of silence’s families, since, as stated by Lope Mateo and Díaz Agea [30], the conspiracy of silence has a strong sociocultural nature, so that neither families nor professionals are not to be held responsible without taking into account their context. That is to say, their motives cannot be considered detached from the social conception of death. In turn, the motives of professionals coincide to a great extent in both processes. Therefore, although the difficulty in communicating bad news and the stereotypes about the illness process do not generally appear in this decision-making process (we believe that this is because there is no concealment of information, as stated above, and because this decision does not have to be taken because of a particular illness), an apparent fear of becoming overinvolved has been observed in both situations. The professionals deny their responsibility in the process, and they point out their fear to take part in aspects that are outside their competencies, which seems to be common and cross-cutting in the professional attitudes in both processes. 

### 4.6. Conflicts Created throughout the Process

There are several problems and conflicts arising from the start of a conspiracy of silence process. They are not only clinical complications and problems, which were mentioned at the beginning of this paper, but they also have an ethical and/or legal dimension, violating the patient’s right to their autonomy [3,12]. This right is protected in Spain by the Law 41/2002, of 14 November, regulating patient autonomy and rights and obligations of information and clinical documentation [34]. However, apart from that, this right is also included as an ethical principle to follow and respect at the professional level [35]. Therefore, the patient has the right both not to be informed and to be informed [1,2,3,5,9,14,36,37], apart from having the right to decide if their family is informed or not [3,14,36]. However, the conspiracy of silence directly and flagrantly violates this principle, as the patient is not informed about their condition.

At the cultural level, the patient seems to be the last one to receive this information [37], the family being the main owner of the information about the patient’s illness [14,38], and, until very recently, at the professional level there has been strong paternalism [38]. In fact, Cejudo López et al. [32] report how sometimes this paternalist relationship was found between the patient-family and the doctor, as the latter looks at the decision-making according to what they would have undertaken if the patient were a member of their family.

On that basis, although the family makes the same mistake as the professionals, they are ultimately the ones responsible for informing the patient about their clinical condition [14]. Thus, a dilemma is presented to professionals: respecting the patient or adapting to the family [9]. Legally and/or ethically the answer is clear, since the patient has the right to know (as long as they want to) [1,2,3,5,9], but it is always preferable not to inform the patient contrary to the family’s opinion, but to try to make them change their mind and agree to communicate; i.e., it is always preferable not to have a conspiracy of silence as such [3], and breaking the silence unilaterally would seriously affect the communication with the family [32]. On account of this, having to protect the patient’s right to information does not relieve of the professional responsibility of trying to also help the family in their own process of overcoming this disagreeable situation and trying to make them change their mind [1,2,3]. It is interesting to consider how the participation of patients in decision-making practically disappears when the conspiracy of silence occurs [32].

Furthermore, in the case of making a decision on the location of care, it seems that there is a contrary situation, at least a priori. In this process, the professionals point at an apparently strong respect for autonomy, which is understandable from the point of view of biomedical ethical principles, as the respect for autonomy is established as a professional obligation and not just an ideal [35]. Thus, they say that they cannot be involved in a decision that is not their responsibility, excluding themselves from it. They avoid a decision that they consider outside their field, as, according to their opinion, it belongs to the family or, in any case, in the professional context it would be in the social/sociomedical sphere. A priori, this attitude could be understood as a deep respect towards the patient’s decision; however, this contradicts the implications of really respecting autonomy, as it is not enough to passively respect it, but they should act in an active respectful way in developing or maintaining the capabilities that make it possible to make decisions autonomously [35]. Therefore, this attitude seems to correspond rather to a “false” respect for the patient’s autonomy, making it possible to observe how this bioethical principle is misinterpreted, using it rather as an excuse to protect oneself from becoming involved in a decision that may have negative consequences. 

In this way, it would seem that the decision is left for the family, believing that they are the ones who must make this decision, which seems to agree with the information reported by Beauchamp and Childress [35]. They stated that there was criticism from some authors about the triumph of autonomy in the field of American bioethics, as they seem to understand that the patient is being compelled to choose, although there might be patients who do not want to make decisions or to have information about their condition [35]. Beauchamp and Childress [35] thus state that making decisions autonomously is a patient’s right, but not a duty [35]. Then it seems that an immeasurable respect for autonomy leads to burdening the affected person or their family with the obligation to decide, turning what should be a right into an obligation. 

Finally, it is interesting to point out a fundamental difference between both processes as far as the information is concerned. While in the conspiracy of silence the professionals are the ones who possess it, also having the responsibility to give it to the patient (the family also being aware of this information), in the decision on the location of care the professionals seem to not always have all the information, and sometimes even none. The family seems to have more information, not on a formal level, but because of their proximity to the older person. This circumstance seems to make the professionals act guided by their personal preferences and experiences, rather than by professional guidelines. This situation is reminiscent of some of the results shown by Cejudo López et al. [32], where the doctor would behave in a paternalistic way with the patient-family, because the doctor would approach the decision-making based on what they would have undertaken if the patient were a member of their family.

### 4.7. Therefore, Is It Possible to Talk about the Conspiracy of Silence in Decision-Making on the Location of Care?

Based on our analysis, it is clear that the decision-making process analyzed herein does not correspond to the classic concept of the conspiracy of silence. However, the conjunction of similarities found is also striking, which makes us wonder if this is a different process, or a variation of it.

In principle, if we take into account Lotra [39]’s work, where, starting from her analysis of the literature about the conspiracy of silence, she infers that a conspiracy occurs whenever an uncomfortable or negative fact be concealed, causing, somehow, a feeling of protection [39], the decision-making process that we analyze in this article would definitely be outside the concept of conspiracy of silence. This is because, even though both share the fact that there is avoidance in a protective way, they differ because a fact is not concealed, but avoided. Although the difference may seem, a priori, minor, it is significant, as concealment involves an active action, while avoidance is passive. 

Otherwise, if we stick just to the more literal meaning of the concept, it is obvious that there is deliberate silence around this decision. According to Sanz Rubiales et al. [40], the original term of conspiracy of silence had the aim of denoting a tacit agreement in which “words are not needed”, in order not to talk about a taboo; what is not talked about is shameful or dangerous and it is only dealt with occasionally and using a handpicked code (Page 51) [40], this definition being a little closer to our results. Nevertheless, this same author points to the term of conspiracy of silence rather matching the concept of collusion in denial in medicine [40]. In this, what is sought is not a lack of information, but that the information that is transmitted to the patient should always be nuanced/biased from a positive point of view [40]. This conception does not agree with the findings, as there is no nuancing in information, nor is it blurred—there is just denial of the evidence presented by facts.

By contrast, the authors of this paper believe that this decision-making process can indeed be framed within what is known as the pact of silence. Although in the literature this term is usually used as a synonym for conspiracy of silence [3], according to the analysis of the concept made by Lemus-Riscanevo et al. [11], silence may occur in two different ways, as a conspiracy or as a pact. The conspiracy of silence usually involves healthcare professionals and family members hiding information from the patient, either completely or partially, whereas in the pact of silence both family and patient, and even healthcare professionals, agree on not talking about the patient’s illness process, despite having this information (in either case, this agreement may be explicit or implicit [11]). The latter concept appropriately matches our decision, since everyone involved evades the decision. They avoid it and, somehow, there seems to be an implicit pact at the social level that avoids interfering in or dealing with this decision, despite being aware of what inaction may entail. A comparative summary of both concepts may be found in Table 3.

### 4.8. Limitations, Strengths, and Future Lines of Research

This study has certain limitations that bear mentioning. The first one would be related with the origin of the results: the qualitative results included here were analyzed as a whole, i.e., the findings show a synthesis of the obtained information in the three studied groups, which made it possible to triangulate the information among the three different populations.

In addition, it is necessary to point out that the comparison that was the object of analysis of this article was conducted between two phenomena of different nature: death and decision-making on the location of care. However, the authors consider that the main interest of this study lies in this comparison, as it has been possible to extrapolate highly studied phenomena and processes to other fields of study, which we believe may help to understand and analyze them more deeply, increasing knowledge and the development of both fields, as well as their clinical approach.

Finally, considering that the qualitative study from which the empirical data were obtained was not conducted with the aim of making this comparison (as the object of analysis was an emerging result of it), it would be interesting if in the future some studies were designed and made, sharing these objects of analysis and comparison, so that it is possible to go deeper and compare results to our findings. We believe that knowing the population’s coping strategies before difficult events is crucial to be able to develop professional and institutional strategies that are really effective to approach this type of situations. 

## 5. Conclusions

The decision-making process on the location of care cannot be exactly extrapolated to the conspiracy of silence phenomenon, as fundamental differences have been found between both processes, being the older person’s active participation in the avoidance of the process, and a lack of a process of information concealment.

However, these processes have also shown many similarities regarding the way an extremely disagreeable fact is faced, such as avoidance and denial. Thus, it was possible to see that the decision on the location of care does not match the conspiracy of silence, but it matches the pact of silence. 

Therefore, this analysis has not only shown the similarities and differences between both phenomena, but it has also revealed the possibility of analyzing the extrapolation to other fields of study of extremely important processes in theory and practice, thus aiding their clinical approach and research.

## Figures and Tables

**Table 1:** Descriptive information about the participants.

Descriptive Information about the Participants	Frequency (%)
**Sex:** - **Female** - **Male**	31 (63.27%) 18 (36.73%)
**Population group:** - **Health and social care professionals** - **Caregivers** - **Older people**	23 (46.94%) 10 (20.41%) 16 (32.65%)
**Area:** - **Rural:** - **Urban:**	17 (34.69%) 32 (65.31%)
**Health and social care professionals**	
**Sex:** - **Female** - **Male**	11 (47.83%) 12 (52.17%)
**Profession:** - **Physicians** - **Nurses** - **Social workers** - **Managerial position**	5 (21.72%) 8 (34.78%) 3 (13.04%) 7 (30.43%)
**Caregivers**	
**Sex:** - **Female** - **Male**	9 (90%) 1 (10%)
**Kinship:** - **Spouse** - **Daughter/son** - **Niece** - **Sister** - **Other**	3 (30%) 4 (40%) 1 (10%) 1 (10%) 1 (10%)
**Older people**	
**Sex:** - **Female** - **Male**	11 (69%) 5 (31%)
**Types of care received:** - **Home care** - **Nursing Home**	13 (81.25%) 3 (18.75%)

Source: prepared by the authors.

**Table 2:** Comparison between the conspiracy of silence phenomenon and the decision on the location of care.

	Conspiracy of Silence	Decision-Making on the Location of Care
***Participants***	- Passive participants: patient, cultural pressure. - Active participants: family, professionals, institutional/organizational context.	- Passive participants: cultural pressure. - Active participants: older person, family, professionals, institutional/organizational context.
***Affected population***	Vulnerable persons, including older people.	Older people and their caregivers.
***Problem that needs to be solved***	Information in the context of palliative care, death.	Decision-making on the location of care.
***Coping processes***	Avoidance coping: - Denial of death - Concealment of information - Patient deception	Avoidance coping: - Denial of the need to make a decisión - There is no concealment of information - Self-deception by everyone involved
***Reasons to undertake it***	Family: protecting the patient and themselves Professionals: difficulty in communication, fear of becoming involved, and stereotypes about the illness process	Older person: denial of the loss of capacity and their caregivers’ exhaustion in order not to change their lifestyles Family: denial of the older person’s deterioration and their own limited capacity to provide care, avoiding breaking the ideal of care Professionals: Fear of becoming involved, acting according to personal criteria
***Conflicts created throughout the process***	Infringement of the ethical principle of respect for autonomy	(False) Respect for autonomy

Source: prepared by the authors on the basis of the analysis of the literature performed.

**Table 3:** Differences between the conspiracy of silence and the pact of silence.

Conspiracy of Silence	Pact of Silence
Family and professionals conceal information from the patient.	Family, patient, and professionals agree on not talking about the issue (despite having the information).

Source: prepared by the authors using information from Lemus-Riscanevo et al. [11].

## Data Availability

Not applicable.
